# Keratinocyte Carcinoma and Photoprevention: The Protective Actions of Repurposed Pharmaceuticals, Phytochemicals and Vitamins

**DOI:** 10.3390/cancers13153684

**Published:** 2021-07-22

**Authors:** Celina Pihl, Katrine Togsverd-Bo, Flemming Andersen, Merete Haedersdal, Peter Bjerring, Catharina Margrethe Lerche

**Affiliations:** 1 Department of Dermatology, Copenhagen University Hospital—Bispebjerg and Frederiksberg, 2400 Copenhagen, Denmark; Katrine.Togsverd-Bo@regionh.dk (K.T.-B.); mhaedersdal@dadlnet.dk (M.H.); Catharina.Margrethe.Lerche@regionh.dk (C.M.L.); 2 Department of Pharmacy, University of Copenhagen, 2100 Copenhagen, Denmark; 3 Department of Clinical Medicine, University of Copenhagen, 2100 Copenhagen, Denmark; 4 Department of Dermatology, Aalborg University Hospital, 9100 Aalborg, Denmark; fan@molholm.dk (F.A.); email@peterbjerring.dk (P.B.); 5 Private Hospital Molholm, 7100 Vejle, Denmark

**Keywords:** cancer, cancer prevention, keratinocyte carcinoma, mechanism of action, photocarcinogenesis, phytochemicals, skin, skin cancer, ultraviolet radiation

## Abstract

**Simple Summary:**

Keratinocyte carcinoma is the most common type of cancer. Sun exposure and ultraviolet radiation are significant contributors to the development of carcinogenesis, mediated by DNA damage, increased oxidative stress, inflammation, immunosuppression and dysregulated signal transduction. Photoprevention involves using different compounds to delay or prevent ultraviolet radiation-induced skin cancer. In this review, we look at new avenues for systemic photoprevention that are based on pharmaceuticals, plant-derived phytochemicals and vitamins. We also investigate the mechanisms underlying these strategies for preventing the onset of carcinogenesis.

**Abstract:**

Ultraviolet radiation (UVR) arising from sun exposure represents a major risk factor in the development of keratinocyte carcinomas (KCs). UVR exposure induces dysregulated signal transduction, oxidative stress, inflammation, immunosuppression and DNA damage, all of which promote the induction and development of photocarcinogenesis. Because the incidence of KCs is increasing, better prevention strategies are necessary. In the concept of photoprevention, protective compounds are administered either topically or systemically to prevent the effects of UVR and the development of skin cancer. In this review, we provide descriptions of the pathways underlying photocarcinogenesis and an overview of selected photoprotective compounds, such as repurposed pharmaceuticals, plant-derived phytochemicals and vitamins. We discuss the protective potential of these compounds and their effects in pre-clinical and human trials, summarising the mechanisms of action involved in preventing photocarcinogenesis.

## 1. Introduction to Photocarcinogenesis 

Keratinocyte carcinoma (KC)—consisting of basal cell carcinomas (BCCs) and squamous cell carcinomas (SCCs)—is the most common cancer worldwide [1]. Carcinogenesis is strongly impacted by sun exposure, demonstrated by 90% of KC cases being associated with ultraviolet (UV) radiation (UVR) exposure [2]. UVR consists of UVA (320–400 nm), UVB (280–320 nm) and UVC (200–280 nm), with only the former two reaching the earth [3]. UVR exposure primarily affects the skin. Here, UVR is almost entirely absorbed by the epidermal cells, inducing adverse effects that contribute to UV-induced carcinogenesis, or photocarcinogenesis.

Chemical prevention of photocarcinogenesis—or chemical photoprevention—involves the administration of compounds that counteract the effects of UVR. This can be achieved by direct absorption or reflection of UV rays—often by topical application—or by targeting the biological effects of UVR systemically. 

**Figure 1:**
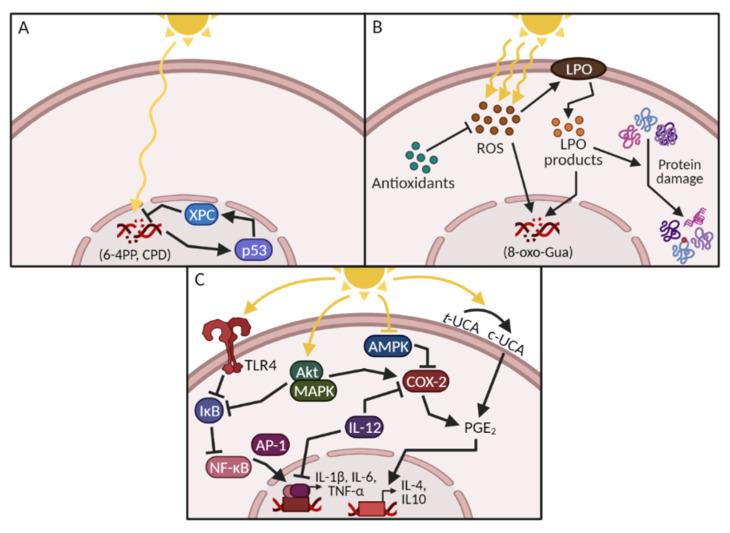
Simplified schematic of (**A**): UV-induced DNA damage caused by direct UV absorption by the DNA molecules resulting in DNA lesions such as 6–4 photoproducts (6–4PPs) or cyclobutane pyrimidine dimers (CPDs) which are repaired by XPC and the nuclear excision repair pathway; (**B**): UV-induced oxidative stress caused by increased reactive oxygen species (ROS) production resulting in damaged DNA (8-oxo-hydroxyguanine lesions), lipid (lipid peroxidation; LPO) and protein (carbonylation and other irreversible changes) molecules; (**C**): UV-induced inflammation, immunosuppression and signal transduction caused by dysregulated mitogen-activated protein kinase (MAPK) activity resulting in activation and transcription of inflammatory mediators. Furthermore, UV radiation induces immunosuppressive mediators via the photoisomersiation of *trans*-urocanic acid (UCA) to *cis*-UCA. Abbreviations: AMPK: AMP-activated protein kinase, AP-1: activator protein 1, COX-2: cyclooxygenase 2, IL: interleukin, PGE_2_: prostaglandin E_2,_ TLR4: Toll-like receptor 4.

In this review, we consider five events induced by UVR that contribute to photocarcinogenesis: DNA damage, oxidative stress, inflammation, immunosuppression and signal transduction (Figure 1A–C).

### 1.1. UV-Induced DNA Damage 

A direct target of UVR exposure is DNA. UVR exposure can result in DNA strand breaks and the formation of 6–4 photoproducts or cyclobutane pyrimidine dimers (CPDs) [4,5]. These lesions are formed in locations with two adjacent pyrimidines and are removed by nucleotide excision repair (NER). In brief, the repair mechanism consists of recognition, incision and replication steps that remove and replace the damaged DNA [6], carried out by the xeroderma pigmentosum family of proteins (XPA–G) (Figure 1A). In particular, XPC reportedly plays a key role in recognising UV-induced DNA damage, as confirmed by mutations and deletions often found in SCC patients [7]. 

**Figure 2:**
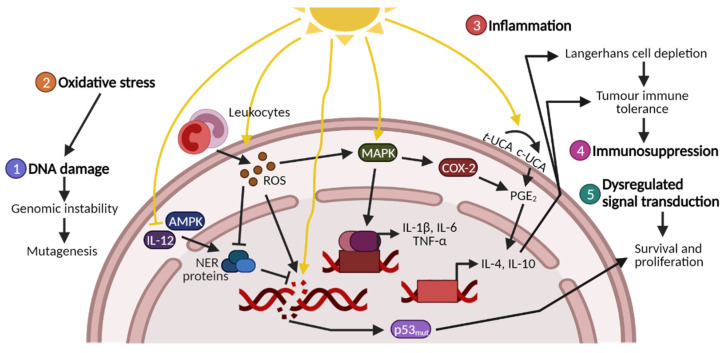
Crosstalk linking UV-induced events: DNA damage, oxidative stress, inflammation, immunosuppression and dysregulated signal transduction as presented in Figure 1. All five events are involved in crosstalk, creating an environment that promotes photocarcinogenesis. Abbreviations: AMPK: AMP-activated protein kinase, AP-1: activator protein 1, COX-2: cyclooxygenase 2, IL: interleukin, MAPK: mitogen-activated protein kinase, NER: nuclear excision repair, PGE_2_: prostaglandin E_2_, ROS: reactive oxygen species, TNF: tumour necrosis factor, *t*(*trans*)/*c*(*cis*)-UCA: urocanic acid.

If the NER pathway does not function correctly and the dimers are not repaired, CC→TT tandem mutations—also called thymine dimers—may be introduced. These are often found in the p53 gene of SCC patients and are recognised as an indicator of UV exposure [8]. Mutations in p53 cause genomic instability and generate a microenvironment that is conducive to tumour development and progression [9]. Together with mutations in other key proteins, this causes an imbalance in tumour suppressor genes and oncogenes, facilitating photocarcinogenesis (Figure 2). 

### 1.2. UV-Induced Oxidative Stress and Protein Damage 

Exposure to UVR increases the production and release of reactive nitrogen and oxygen species (ROS) [10,11]. Although the skin contains several endogenous antioxidants that can counteract endogenous ROS production (Table 1), the increased release of pro-oxidants overwhelms the system and depletes the antioxidant capacity [12]. Thus, the reactive species remain unchecked, causing oxidative stress to DNA, lipid and protein molecules (Figure 1B).

**Table 1:** Non-exhaustive list of reactive species generated by UV radiation and endogenous antioxidants found in the skin.

Reactive Species		Antioxidants	
Hydrogen peroxide (H_2_O_2_)	Hydroperoxyl (HO_2_)	Catalase	Glutathione
Hydroxyl radical (OH)	Nitric oxide (NO)	Glutathione peroxidase	Superoxide dismutase
Singlet oxygen (^1^O_2_)	Superoxide (O^–^_2_)	Vitamin C	Vitamin E

Guanine nucleobases in the DNA are particularly sensitive to oxidation [13]. The introduction of oxidised bases such as 8-hydroxyguanine (8-oxo-Gua) may cause G:C→T:A transversions which, together with other DNA lesions and impaired NER components, enhance the mutagenic potential of UV exposure [14,15]. 

Lipid peroxidation (LPO) represents the major oxidative damage affecting lipids. Here, ROS attack the allylic carbon atom of a lipid molecule, often polyunsaturated fatty acids, and abstract the hydrogen molecule, creating a lipid radical. The lipid then reacts with oxygen to form a lipid peroxyl radical which, in turn, can abstract hydrogen molecules from nearby lipids, initiating a chain reaction and forming new radicals [16]. During homeostasis, a termination step follows in which an antioxidant, often vitamin E, donates a hydrogen molecule to the lipid peroxyl radical to stop further reactions. Vitamin E is then reduced via a cycle involving vitamin C, glutathione and NAD(P)H, restoring its antioxidant activity [17]. However, as the ratio of antioxidants to ROS is reduced during UVR exposure, the LPO reactions continue to cause damage. 

One target of LPO is the phospholipids in the cell membranes. Damage impairs membrane integrity, which affects the activity of membrane-bound proteins and contributes to a pro-apoptotic state [18]. Secondary aldehyde products of LPO, such as malondialdehyde and 4-hydroxynonenal, can also react with thiol and amino groups, leading to changes in protein activity, binding or turnover as a result of altered side chains, aggregation, fragmentation or conformational changes [19,20]. In addition to direct interaction with nucleosides to form adducts, malondialdehyde also inhibits the NER pathway, leaving cells vulnerable to UV-induced mutagenesis (Figure 1B and Figure 2). 

Another effect of oxidative stress is protein carbonylation. Here, reactive carbonyl groups—e.g., originating from aldehydes—are introduced into proteins via oxidation of amino acid side chains [21]. This modification is irreversible and, because carbonylated proteins are prone to aggregation, these proteins must be degraded [22]. Finally, ROS can mimic the effects of tumour promoters, which contributes to the transformation of cells [23].

### 1.3. UV-Induced Inflammation, Immunosuppression and Signal Transduction 

UVR exposure elicits a prolonged inflammatory response that presents as erythema, hyperplasia and oedema caused by increases in vascular flow and permeability. This is triggered by the release of inflammatory mediators such as cytokines and chemokines that recruit leukocytes to the irradiation site [24]. These cells contribute to the inflammatory state which also stimulates ROS production, thereby increasing oxidative stress (Figure 2). 

One of these mediators is the transcription factor NF-κB, which is sequestered in the cytosol by the inhibitory protein IκB. Upon UV exposure, IκB is targeted for degradation via mitogen-activated protein kinase (MAPK) signalling, facilitating NF-κB’s nuclear translocation to regulate target gene expression [25,26]. In particular, NF-κB regulates the expression of the pro-angiogenic permeability factor vascular endothelial growth factor, as well as interleukin (IL)-1β, IL-6 and tumour necrosis factor (TNF-α), all of which are implicated in photocarcinogenesis (Figure 1C) [27]. 

Another marker of UV-induced inflammation is the upregulation of cyclooxygenase (COX)-2 expression induced by ROS and the MAPK pathways [28]. The COX enzymes include the constitutive COX-1 and the inducible COX-2 forms. The COX enzymes stimulate the synthesis of prostaglandins (PGs), where PGE_2_ is produced abundantly in the skin. PG-stimulated signalling promotes angiogenesis, infiltration of leukocytes at the irradiation site and cellular proliferation [29,30]. 

UVR exposure also reportedly acts immunosuppressive. Here, UV exposure targets the antigen-presenting Langerhans cells, impairing their function. UVR exposure reduces the expression of MHC II and co-stimulatory molecules, thereby preventing antigen presentation [31,32]. Another sign of chronic UVR exposure is Langerhans cell depletion, either via migration to lymph nodes or—when UV doses are high—apoptotic cell death [33]. 

Trans-urocanic acid (UCA) is a histidine metabolite located in the skin, where it provides endogenous protection by acting as a photoreceptor. Upon UV exposure, trans-UCA is photoisomerised to cis-UCA, inactivating its photoprotective properties [34,35]. Furthermore, cis-UCA also induces the release of PGE_2,_ which activates a cascade of suppressive cytokines resulting in the expression of IL-4 and IL-10 (Figure 1C and Figure 2) [36,37]. IL-10 is a key immunosuppressive mediator involved in impairing the antigen-presenting cell (APC) function of Langerhans cells [38], as well as preventing other APCs from stimulating Th-cells [39]. 

UVR induced immunomodulation presents a dynamic range of cellular states from inflammatory to immunotolerance to immunosuppression, creating an environment that supports and promotes carcinogenesis [40]. 

Whether it is UV-induced DNA damage, oxidative stress, inflammation or immunosuppression, signal transduction plays an important role in providing the optimal conditions for tumour development. As with many other cancer types, signal transduction during photocarcinogenesis is heavily impacted by MAPK signalling via p38, JNK and ERK and the subsequent activation of NF-κB and activator protein 1 (AP-1), as well as the inactivation of AMP-activated protein kinase (AMPK) (Figure 1C, Figure 2) [41,42]. 

### 1.4. Photocarcinogenesis and Photoprotectants

During photocarcinogenesis, UV-induced DNA damage, oxidative stress, inflammation, immunosuppression and dysregulated signal transduction all participate in crosstalk as part of the initiation, development and progression of skin cancer (Figure 2). Therefore, all five categories must be evaluated to identify potential photoprotectants. 

A compound’s photoprotective potential is usually evaluated in skin-derived cell and mouse models with human trials later on when the photoprotective potential has been established. The immunocompetent hairless mouse model is frequently used in these studies as tumour development can be monitored throughout the intervention period [43].

In this review, we have included five different categories of compounds from pharmaceuticals, phytochemicals and vitamins that have exhibited photoprotective properties. The purpose of this strategy was to strike a balance between a comprehensive description of a given compound with its mechanism of action and discussing the diverse effects of compounds within these three groups. These fifteen categories have been included because they each represent a characteristic photoprotective mechanism. Consequently, we illustrate specific aspects of how to target UV-induced effects using candidate compounds and we simultaneously provide insight into the properties required to prevent skin cancer. A range of studies is described, from initial testing in mouse models to year-long human trials. This approach emphasises the progression within this research field as well as the compounds that have emerged as potential photoprotectants. In Table 2, Table 3 and Table 4, we provide an overview of the various protective mechanisms and present results from photocarcinogenesis prevention studies in humans and mouse models.

## 2. Pharmaceuticals: From Repurposing to Prevention 

### 2.1. Non-Steroidal Anti-Inflammatory Drugs 

Some of the most frequently prescribed drugs worldwide are the non-steroidal anti-inflammatory drugs (NSAIDs). These are commonly used as painkillers for numerous conditions because of their analgesic and anti-inflammatory properties. NSAIDs interfere with PG-synthesis through inhibition of the COX enzymes [44]. COX-2 induction is an important mediator of photocarcinogenesis. Therefore, COX-2 specific and non-specific NSAIDs presented as an attractive source for photoprotection. However, as the field has progressed, pharmaceutical photoprevention has increasingly focused on repurposing drugs with anticarcinogenic properties and evaluating whether these drugs could be used in photoprotection. NSAIDs such as celecoxib, nimesulide, indomethacin and acetylsalicylic acid have reportedly exhibited protective effects during in vivo studies. These NSAIDS delayed tumour onset and reduced tumour incidence, size and progression, as well as triggering tumour regression in some cases [45,46]. The anti-tumour effects were accompanied by reduced DNA damage and inflammation, decreased MAPK, NF-κB and COX-2 activation and PGE_2_ synthesis [47,48,49]. 

Consequently, NSAIDs were tested in clinical trials. In an 11-month clinical trial, administration of 400 mg celecoxib daily to subjects with premalignant actinic keratoses (AKs) reduced the total number of KCs as well as BCCs and SCCs individually [50]. In another study, topical application of 1% piroxicam—a non-specific NSAID—triggered regression in 48% of AKs included in the study (Table 2) [51]. 

However, although NSAIDs have demonstrated photoprotective potential in both mouse and human studies, other studies have described the potentially adverse side effects of prolonged NSAID use. These include increased risk of cardiovascular events [52], gastrointestinal bleeding [53] and renal failure [54]. 

### 2.2. AMPK Activators: Metformin 

Increasing evidence has implicated AMPK as a potential target for cancer therapies [55]. AMPK as an energy and nutrient sensor can interact with p53 via metabolic checkpoints to induce cell cycle arrest [56,57]. Additionally, tumours from UV-irradiated human and murine skin display decreased AMPK activation [41], suggesting a role for AMPK activation in photoprotection. Metformin is commonly used to treat diabetes by inhibiting protein kinase A and activating AMPK, leading to decreased gluconeogenesis in the liver and increased insulin sensitivity in target tissues [58]. Interestingly, metformin treatment is reportedly associated with a 31% decrease in overall cancer risk, compared to other antidiabetic treatments [59]. 

In keratinocytes, incubation with metformin protected against UV-induced inflammation, as demonstrated by impaired NF-κB activity and reductions in IL-1β, IL-6 and TNF-α expression [60]. Similarly, a study on nude mice with SCC A431 tumour-cell xenografts reported impaired NF-κB activity as well as reduced COX-2 expression following injection with metformin [61]. 

Metformin reduced ROS formation and expression of matrix metalloproteinase (MMP) 1 and 3 in vitro [62]. During homeostasis, MMPs partake in remodelling and degradation of the extracellular matrix [63]. However, MMPs can also stimulate tumour development and angiogenesis. Furthermore, topical application of metformin in hairless mice increased CPD repair six hours after UVR exposure [41]. In xenografted mice, injection with metformin also protected against UV-induced proliferation, inducing apoptosis in the tumours along with reductions in protein kinase B (Akt), MAPK and NF-κB signalling [61]. Moreover, metformin may specifically target the skin’s cancer stem cell diaspora [64,65], further illustrating its photoprotective potential. 

In hairless mice, topical and oral administration of metformin delayed tumour onset, decreased tumour multiplicity and volume and stimulated DNA repair (Table 2) [41]. In vitro studies using AMPK-knockout cells reported that metformin-induced DNA repair was dependent on AMPK activation [41]. 

There have been no clinical studies on the effect of metformin on KCs. However, a population-wide study in Taiwanese diabetic patients reported a significantly lower risk of skin cancer incidence in metformin-treated subjects compared to those who had never received metformin [66]. Another recent study across the Icelandic population found a significantly lower risk of BCCs, but not SCCs, following metformin use [67]. 

Metformin, therefore, represents a promising candidate for photoprotection, but more studies are needed, namely clinical trials, to investigate its effects. Furthermore, AMPK activators such as 5-aminoimidazole-4-carboxamide ribonucleotide (AICAR) [41] and phenformin [68,69] should also be investigated as potential photoprotectants.

### 2.3. Toll-Like Receptor 4 Antagonism: Resatorvid 

Because inflammation is an important event in the development of photocarcinogenesis, some recent research has focused on targeting immunomodulators such as the Toll-like receptors (TLRs). In particular, TLR4 has been thought to be a driver in cutaneous inflammation activating MAPK, AP-1 and NF-κB, as well as the expression of IL-1β, IL-6 and TNF-α [70] (Figure 1C).

In the skin, TLR4 expression is upregulated in keratinocytes following UVR exposure [71]. A study that compared normal skin, sun-damaged skin and AKs from the same individuals reported that TLR4 expression was confined to the basal layer of the epidermis in normal skin, whereas in response to sun damage, TLR4 was strongly expressed across several epidermal layers, with thicker and more pronounced detection in the AK samples [72]. Therefore, TLR4 antagonism is an emerging target for limiting UV-induced inflammation. 

Resatorvid, or TAK-242, is a small-molecule inhibitor that selectively binds to TLR4 and inhibits cellular activity by preventing TLR4 from interacting with adaptor molecules [73]. Resatorvid has been reported to have neuroprotective effects following brain injury [74], and is currently under investigation for its anticarcinogenic properties demonstrated in breast and ovarian cancer cell lines in which TLR4 antagonism reduced epithelial–mesenchymal transition and invasion [75]. 

In a study on irradiated keratinocytes, incubation with resatorvid prevented activation of p38, JNK and Akt. This was accompanied by reductions in AP-1 and NF-κB activity and IL-6 and IL-8 expression [72]. Notably, the study reported that incubation with resatorvid pre- or post-UVR had similar effects, suggesting that these are not caused by UV absorption but by resatorvid’s antagonism of TLR4. 

Irradiated hairless mice treated with a topical formulation of resatorvid also demonstrated reduced activity of the MAPKs, AP-1 and NF-κB with decreased expression of IL-6, IL-8 and IL-10 [72,76]. When photocarcinogenesis is stimulated experimentally, topical application of resatorvid delayed tumour onset and reduced tumour multiplicity and incidence in hairless mice [76]. Interestingly, this study reported that only when resatorvid was used as a prevention measure—i.e., administered together with UV—and not an intervention treatment—i.e., resatorvid given after UV was terminated—were there reductions in tumourigenesis (Table 2). 

Resatorvid has been tested in a clinical trial as a treatment for septic shock. Although it failed to suppress patients’ cytokine levels, resatorvid was generally well tolerated [77]. Therefore, resatorvid is a promising candidate for photoprotection, but more studies are needed to elucidate its mechanism of action, determine whether the protective effects can be translated to humans and establish a suitable therapeutic window. 

### 2.4. Oestrogen Receptor Signalling: Erb041, 17β-Oestradiol and Phytoestrogens 

Another potential pharmaceutical target for skin cancer prevention is the modulation of the oestrogen receptors (ERs)—ERα and ERβ. Both receptors are activated by oestrogen (17β-oestradiol) and related oestrogenic compounds, resulting in target gene transcription. Despite sharing ligands, the two receptors often act antagonistically, with ERβ reportedly functioning as a tumour suppressor to prevent tumour metastasis and proliferation induced by ERα [78,79]. ERβ specific agonists have been tested in clinical trials to promote the anticarcinogenic effects of ERβ without stimulating ERα signalling. 

Erb-041, or Prinaberel, is an oestrogenic ligand with a similar binding affinity to oestrogen but optimised to selectively bind to ERβ [80]. A clinical trial monitored Erb-041 intake for its effects on rheumatoid arthritis over a 12-week period, and although treatment failed to show efficacy in patients, the study did report that Erb-041 was well-tolerated and exhibited a good safety profile [81]. In the skin, ERβ expression is normally confined to the basal layer of the epidermis. This expression is reduced in tumour adjacent skin and lost entirely in skin tissue derived from SCCs. Moreover, ERβ expression is lost in UV-exposed murine skin [82], further suggesting that ERβ signalling is a potential target for skin cancer prevention. 

One study assessed the effect of Erb-041 in preventing photocarcinogenesis. Treatment with Erb-041 restored ERβ expression in both irradiated murine skin and cell cultures, and topical application of Erb-041 to hairless mice led to a delay in tumour onset. The mice in this study also exhibited reductions in tumour incidence, multiplicity and volume as well as in carcinoma progression. This was accompanied by a decreased inflammatory response manifesting as reduced leukocyte infiltration, hyperplasia and inflammatory cytokines. Furthermore, Erb-041 application resulted in reduced activation of ERK, p38, NF-κB and Akt signalling and decreased expression of proliferation, angiogenesis and epithelial-mesenchymal transition markers [82]. These observations suggest that Erb-041 can prevent UV-induced events (Table 2). 

Other non-specific oestrogenic compounds have also been tested as photoprotectants. For example, 17β-oestradiol injections reduced UV-induced immunosuppression and associated IL-10 production in male mice [83]. Some phytochemicals also have oestrogenic properties, as described below (Section 3). These natural phytoestrogens share structural similarities with oestrogen and are capable of modulating ER signalling, albeit with lower potency [84]. Phytoestrogens such as genistein, epicatechin and resveratrol exhibit numerous protective effects [85]; the latter two are discussed in Section 3.1and Section 3.2.

Although activation of ERβ signalling is a potential target for preventing UVR-induced effects, more studies are needed to understand how ERβ signalling operates photoprotection. As administration of oestrogenic compounds may also result in some side effects, it is possible that the less potent phytoestrogens may be suitable alternatives. 

### 2.5. Recent Discoveries in Pre-Clinical Studies: Carvedilol and Bucillamine 

Finally, carvedilol and bucillamine have been studied over the past five years as potential photoprotectants. 

Carvedilol is a β-adrenergic receptor (β-AR) antagonist that prevents the binding of catecholamines such as nor- and epinephrine to the β-AR, licenced for treatment of hypertension and heart disease. However, catecholamines also reportedly impact carcinogenesis by affecting DNA repair and APCs [86,87]. As keratinocytes express β-AR-2 [88], carvedilol was tested in epidermal cells and hairless mice for its potential in photocarcinogenesis prevention. 

In vitro studies reported that following carvedilol treatment, irradiated cells exhibited reductions in NF-κB and AP-1 activity, PGE_2_ release and colony formation [89,90], whereas topical application in reconstituted human skin reduced inflammation markers such as COX-2 and TNF-α expression, as well as hyperplasia and apoptosis [91]. Furthermore, carvedilol treatment decreased inflammatory cytokines and CPDs in hairless mice when photocarcinogenesis is stimulated experimentally. These mice also exhibited delays in tumour onset and reduced tumour multiplicity and incidence [90].

Bucillamine is a cysteine-derived compound that contains two thiol groups, which confer significant antioxidant activity. Bucillamine has been used for more than 30 years in Japan and South Korea as a treatment for rheumatoid arthritis and is well tolerated [92], but it is not licenced for use in the European Union. 

Bucillamine shares structural similarities with N-acetylcysteine, a proven photoprotectant in hairless mice [93], but it is reportedly even more potent as an antioxidant [94]. Therefore, the photoprotective potential of bucillamine was evaluated. In irradiated keratinocytes and hairless mice, treatment with bucillamine reduced the activation of JNK and caspase 3 [95]. Furthermore, the mice exhibited reductions in dermal oedema, leukocyte infiltration, proliferation and p53 expression [95,96]. These results indicate that bucillamine treatment can prevent UV-induced skin damage in hairless mice. Therefore, future studies should investigate its effects on photocarcinogenesis, focusing on whether bucillamine’s inherent antioxidant activity can limit the impact of oxidative stress. 

To sum up, the use of pharmaceuticals in photoprotection is an evolving field. Whereas previously, much research focused on preventing the induction of COX-2 by implementing specific inhibitors (NSAIDs), recent studies have involved drug repurposing to avoid the expensive and time-consuming clinical trials necessary to evaluate novel pharmaceuticals. In this section, we have discussed a selection of compounds that may not be ready to use as treatments but do highlight events and mechanisms that may be targeted in future studies. In Table 2, we have provided an overview of the seven compounds discussed in this section, their proposed mechanisms and potential (pre-)clinical results in human and mouse studies.

**Table 2:** Selected pharmaceuticals that have been tested for photoprotective effects, their proposed mechanisms and, if applicable, (pre-)clinical results.

Compound	Model	Mechanism of Action	(Pre-)Clinical Results	Ref
17β-oestradiol	Mice	Activates ER signalling, and reduces immunosuppression		[83]
Bucillamine	Hairless mice, keratinocytes	Reduces proliferation, cell cycle arrest and apoptosis while preventing leukocyte infiltration.		[95,96]
Carvedilol	Hairless mice, epidermal cells, ex vivo skin	Induces DNA repair while reducing the inflammatory response via AP-1 and NF-κB inhibition.	Mice: Delays tumour onset and reduces tumour incidence and multiplicity.	[89,90,91]
Erb-041	Hairless mice, keratinocytes	Activates the ERβ receptor which inhibits proliferation, angiogenesis and EMT in tumour tissue. Reduces signal transduction and the inflammatory response.	Mice: Delays tumour onset, reduces tumour incidence, volume and multiplicity and prevents SCC progression.	[82]
Metformin	Hairless mice, Xenografted (A431) mice, keratinocytes	Activates AMPK signalling which facilitates DNA repair, reduces the inflammatory response and induces tumour cell apoptosis.	Mice: Delays tumour onset and reduces tumour incidence and multiplicity.	[41,60,61]
NSAIDs	AK-affected individuals, hairless mice	Prevents DNA damage, reduces COX-2 induction and the inflammatory response via AP-1 and NF-κB inhibition.	Human: Reduces keratinocyte carcinomas (SCCs and BCCs) and promotes AK regression.Mice: Delays tumour onset and reduces tumour incidence, progression and multiplicity.	[47,48,49]
Resatorvid	Hairless mice, keratinocytes	Inhibits TLR4 signalling which reduces MAPK, AP-1 and NF-κB signalling and the inflammatory response.	Mice: Delays tumour onset and reduces tumour incidence and multiplicity.	[72,76]

Abbreviations: AK: actinic keratosis, AMPK: AMP-activated protein kinase, AP-1: activator protein 1, BCC: basal cell carcinoma COX-2: cyclooxygenase, ER: oestrogen receptor, EMT: epithelial-mesenchymal transition., MAPK: mitogen-activated protein kinase, NSAIDs: non-steroidal anti-inflammatory drugs, SCC: squamous cell carcinoma, TLR4: Toll-like receptor 4.

## 3. Dietary and Non-Dietary Phytochemicals 

Whereas the use of pharmaceuticals in photoprotection has primarily focused on anti-inflammatory drugs and possible repositioning strategies, the use of plant-derived compounds—referred to as phytochemicals—has shown great potential for photoprotection. Phytochemicals are diverse compounds that can modulate oxidative stress, inflammation, signal transduction and other pathways that contribute to photocarcinogenesis [97]. Polyphenols are particularly common photoprotective phytochemicals that act as antioxidants to prevent ROS formation. 

### 3.1. Green Tea and Polyphenols 

The polyphenols from green tea (*Camellia sinensis*) have been studied intensively for their effects on photocarcinogenesis. The focus has been on epigallocatechin-3-gallate (EGCG)—the most abundant polyphenol found in green tea—because it exhibits the greatest activity [98]. Incubation with green tea polyphenols (GTPs) increases antioxidant activity and reduces the release of ROS in vitro [99,100]. Furthermore, GTPs reduce the phosphorylation and activation of MAPKs and NF-κB [100,101]. 

Similar effects have been reported in hairless mice in which GTP administration also led to reductions in tumour incidence, multiplicity and malignant transformation, as well as delays in tumour onset [102,103]. Mice receiving oral administration of GTPs also exhibited reduced levels of inflammation and PGE_2_ and decreased expression of IL-1β, IL-6, TNF-α and COX-2 [103]. Moreover, topical GTP application reduced UV-induced oedema, hyperplasia and infiltration of leukocytes (Table 3) [102]. 

Mechanisms have been proposed to explain these protective effects. EGCG-treated irradiated mice exhibited decreased levels of DNA hypomethylation due to downregulation of DNA methylases [102], which otherwise can mediate malignant transformation via transcriptional changes [104]. GTP may also stimulate photoprotection via IL-12, which counteracts DNA damage by increasing NER-gene expression (Figure 2) [105]. EGCG induced IL-12 in keratinocytes, whereas subsequent addition of anti-IL-12 antibodies eliminated the protective effects of EGCG [106]. Similarly, GTP treatment has less effect on IL-12-KO mice than on wild-type mice [103], indicating that IL-12 is important for this protective effect. 

GTPs have been tested both topically and orally in human trials, with conflicting results. Topical application of GTPs provided protection against UV-induced DNA damage and erythemal response [107], whereas oral administration of 800 mg or 1080 mg of EGCG provided no protection [108,109]. Similar results were reported for oral administration of 540 mg GTPs and 50 mg vitamin C over a period of three months [110]. This discrepancy is surprising as GTPs reportedly have no significant UV-absorption that could potentiate its topical effect. 

Despite the promising in vivo studies, the results from human trials were disappointing. Furthermore, GTPs are prone to oxidation and degradation [111] and have a half-life of only five hours in human circulation [112], restricting effective administration and storage. 

### 3.2. Grapes and Related Polyphenols: Proanthocyanidins, Resveratrol and Pterostilbene 

Grapes and their seeds contain polyphenols, such as proanthocyanidins and oestrogenic stilbenes such as resveratrol. Incubating various skin cell models with grape extracts has yielded promising results, including reductions in DNA damage, inflammation and oxidative stress [113], and several studies have explored these protective effects in the hairless mouse model [114,115,116,117,118]. 

Dietary supplementation to mice undergoing UVR with grapes or grapeseed extracts had anticarcinogenic effects. These mice exhibited increased antioxidant capacities, manifesting as decreases in LPO, protein carbonylation and ROS [116]. MAPK and NF-κB activation were also impaired following oral intake. This was accompanied by reductions in UV-induced inflammation, demonstrated by decreases in cutaneous leukocyte infiltration, PGE_2_ release and expression of COX-2 as well as the inflammatory cytokines IL-1β, IL-6 and TNF-α [114]. IL-10 expression was also reduced, indicating decreased immunosuppression [117]. 

Grape constituents may also counteract tumour formation and development. Providing hairless mice with grape-based supplements reduced proliferation markers and CPD formation facilitated by increased NER-genes transcription [114,118]. This was reflected by reductions in tumour incidence, multiplicity, size and progression [114,115]. 

Similar effects have recently been observed in human trials. For example, Oak et al. reported that a daily intake of 75 g freeze-dried grape supplement—containing both proanthocyanindins and resveratrol—for 14 days led to an increase in mean minimal erythema dose (MED) pre- and post-intervention (173.1 and 267.6 J/m^2^, respectively) [119]. Oak et al. also demonstrated reductions in epidermal apoptosis, DNA damage and downregulation of inflammatory mediators such as IL-1β, IL-8 and IL-22 following intake of a dietary grape supplement [120]. 

These reported effects of grape constituents suggest that pterostilbene may also have promising protective effects, although few relevant studies have specifically investigated photoprotection. Pterostilbene is a dimethylether analogue of resveratrol and reportedly has 3–4 times the bioavailability [121]. Irradiated hairless mice treated with a topical pterostilbene cream exhibited reduced signs of skin damage such as wrinkling and hyperplasia. The mice also had increased antioxidant capacity with subsequent decreases in LPO, protein carbonylation and oxidative DNA lesions. Finally, the study concluded that the application of pterostilbene but not resveratrol reduced tumour incidence in these mice (Table 3) [122].

Based on these results, more research should focus on elucidating the effect of grapes in human photoprotection. Furthermore, studies should explore whether the increased bioavailability of pterostilbene and the improved in vivo results can be translated into clinical results to provide an additional avenue for protection. 

### 3.3. Polypodium Leucotomos

Polypodium leucotomos (PL) is a fern plant with a high content of polyphenols native to Central and South America [123]. An aqueous extract of the leaves containing these phenols is sold as a dietary supplement under the tradename Fernblock^®^. Fernblock^®^ and other extracts of PL have exhibited photoprotective properties both in vitro and in vivo, as well as in clinical trials. 

The mechanism of photoprotection has been investigated extensively and involves significant antioxidant activity. PL provided protection against ROS formation and LPO in vitro [124,125], whereas oral administration of PL to hairless mice reportedly increased antioxidant enzyme capacity and prevented the formation of oxidative DNA lesions [126,127]. These mice also exhibited reductions in CPD formation and proliferation markers with increased p53 expression. 

PL had immunomodulatory activity and decreased transcriptional activation of NF-κB and AP-1, as well as expression of TNF-α in vitro [128]. In hairless mice, PL counteracted COX-2 induction and infiltration of leukocytes following UVR exposure [127]. It also prevented immunosuppression [129], as demonstrated by the inhibition of photoisomerisation and photodecomposition of trans-UCA [124], as well as inhibition of Langerhans cell depletion in hairless rats [130]. 

Oral administration of PL to hairless mice significantly delayed tumour onset and decreased the incidence of AKs following UVR exposure [131]. Decreased tumour incidence was also reported in a similar study (Table 3) [132]. 

In healthy volunteers, a dietary supplement (1080 mg) or topical application (10%) of PL prior to UVR exposure significantly increased the MED (80, 98 and 34 mJ/cm^2^ for dietary administration, topical administration and untreated controls, respectively) and prevented depletion of Langerhans cells [133]. Studies with a lower dietary dose of 240 mg reported similar protective effects including reductions in erythema and oedema [134,135], sunburn cell formation, CPDs [134,135,136], expression of COX-2 and proliferation markers [136], as well as inhibition of Langerhans cell depletion [134]. 

Furthermore, 240 mg of PL taken twice a day for two months provided effective protection against sun damage evaluated by MED and erythema with no adverse side effects [137]. Overall, PL is a promising photoprotectant that decreases tumourigenesis in mouse models and counteracts harmful acute UV-induced effects in humans. However, further studies are needed to elucidate the long-term effects of PL on skin cancer development.

### 3.4. Berries: Pomegranate, Raspberries and Blackberries 

Because of the potential impact of polyphenols in photoprotection, the properties of berries also warrant investigation. Berries such as pomegranate, raspberries and blackberries have very high polyphenolic contents and are easy to purchase.

Among these three, the anticarcinogenic properties of pomegranate have been studied most extensively. Pomegranates from the Punica granatum tree contain antioxidants that are more potent than those from sources such as red wine and green tea [138]. In hairless mice, a dietary supplement of pomegranate extract decreased MAPK signalling, inhibited NF-κB activity and led to reductions in the inflammatory response, including decreased COX-2 expression and leukocyte infiltration [139]. Furthermore, oral administration to mice reduced CPDs, oxidative DNA lesions and other markers of oxidative stress [140]. 

When photocarcinogenesis is stimulated experimentally, pomegranate-fed mice exhibited a decreased incidence of SCCs with reductions in both p53 expression and proliferation [141]. In human studies, intake of pomegranate over 12 weeks provided protection against UVR, as shown by increases in MED from baseline to post-treatment [142]. 

Although there have been no clinical trials, raspberries also have great potential as a photoprotectant. Hairless mice treated topically with a black raspberry extract following UVR exposure exhibited decreases in tumour multiplicity and size. This was accompanied by reductions in the number of 8-oxo-Gua lesions and in p53 expression, as well as a reduced inflammatory response, which was indicated by decreased neutrophil activation and oedema [143]. Recent studies have also shown that red raspberries exhibit promising topical photoprotectant activity. Albeit no data on tumour formation, irradiated mice treated with a red raspberry extract demonstrated reductions in erythemal response, p38, AP-1 and NF-κB activity, together with decreased COX-2 expression. The antioxidant capacity was also increased in these mice, possibly via nuclear factor erythroid 2-related factor (Nrf-2) activation, which induces transcription of antioxidant-response genes and decreases 8-oxo-Gua lesions and protein carbonylation [144], mimicking results reported in vitro [145]. 

Finally, blackberries and one of their polyphenols, cyanidin-3-glucoside, have displayed promising photoprotective characteristics in vivo. Topical application of blackberry extract or the polyphenol to hairless mice undergoing UVR showed reductions in both the inflammatory and oxidative stress responses. These mice exhibited reductions in LPO and oxidative DNA lesions, as well as decreased oedema, hyperplasia and leukocyte infiltration. Reductions in MAPK signalling and NF-κB activity were also reported, together with subsequent decreases in PGE_2_ release and expression of iNOS, IL-6 and TNF-α (Table 3) [146,147]. 

Although some of these studies focused on topical application, the widespread availability of these berries is ideal for oral photoprotection. However, further studies are needed to confirm the protective potential of berries: extensive mouse studies should be used to elucidate protective mechanisms and clinical trials are needed to investigate whether berries can prevent KCs in humans. 

### 3.5. Cocoa Flavanols 

Polyphenolic compounds can also be found in cocoa as so-called cocoa flavanols. Although chocolate and other cocoa-containing products may have a high cocoa content, the flavanols are often destroyed in processing steps before the final products are generated. Nevertheless, the potential of cocoa extracts in epidermal health has been suggested by pointing to effects such as ROS scavenging, prevention of MAPK and NF-κB activation and inhibition of COX-2 induction [148,149]. 

Because similar pathways contribute to KC development, the effect of cocoa extract was tested in hairless mice when photocarcinogenesis was stimulated experimentally. Following oral intake of cocoa extract, these mice had fewer wrinkles and MMP-1 expression was downregulated [150]. Irradiated mice given cocoa extract also exhibited a lower incidence of invasive SCCs, as well as decreases in mutated p53 expression and PGE_2_ release [141], indicating increased protection against UV-induced events and carcinogenesis. 

Cocoa extract has also proved beneficial in humans. In 2006, a clinical trial spanning over 12 weeks reported that a daily intake of 329 mg cocoa flavanols reduced UV-induced erythema by 25%, whereas a lower intake (27 mg) did not [151]. Other studies exploring shorter and longer intervention periods (1 and 24 weeks, respectively) had similar outcomes, resulting in increases in MED [150,152]. However, two studies investigated the effects of a higher dose (600 mg) over 12 weeks, and only one of these studies found a protective effect [153,154], suggesting that more research is needed to clarify the photoprotective effects of cocoa flavanols. 

As described in this section, dietary and non-dietary phytochemicals have enormous potential for photoprotection. Pre-clinical and human trials have shown that these compounds have antioxidant, anti-inflammatory and anti-carcinogenic effects in subjects exposed to UVR (Table 3). 

In contrast to pharmaceuticals, which are expensive and may have adverse side effects, phytochemicals are widely available and may be readily included in the daily diet. Furthermore, while phytochemical intake at higher concentrations may have some side effects, they are not as severe as what is demonstrated with certain pharmaceuticals. However, because some polyphenolic compounds absorb UVR, observed effects may be due to a sunscreen effect leaving oral administration inefficient [155]. Therefore, more research is needed to evaluate the use of phytochemicals in long-term systemic photoprevention. These studies should focus on dosing, delivery and effectiveness to optimise the protective potential.

**Table 3:** Selected phytochemicals that have been tested for photoprotective effects, their proposed mechanisms and, if applicable, (pre-)clinical results.

Compound	Model	Mechanism of Action	(Pre-)Clinical Results	Ref
Blackberries	Hairless mice, keratinocytes, ex vivo skin	Reduces oxidative stress and the inflammatory response.		[146,147]
Cocoa flavanols	Healthy volunteers, hairless mice	Reduces mutagenesis of p53, inflammatory markers and degradation of the extracellular matrix.	Mice: Reduces invasive SCCs.	[141,150,151,152,153,154]
Grape seeds	Healthy volunteers, hairless mice, keratinocytes	Increases antioxidant activity, reduces the inflammatory response and proliferation and promotes DNA repair.	Mice: Reduces tumour incidence, size, multiplicity and progression.	[114,115,116,117,118,119,120]
Green tea	Healthy volunteers, hairless mice, keratinocytes	Prevents DNA hypomethylation, stimulates IL-12 (facilitating DNA repair) and reduces the inflammatory response and oxidative stress.	Mice: Delays tumour onset and reduces tumour incidence, multiplicity and progression.	[99,100,101,102,103,106]
Polypodium leucotomos	Healthy volunteers, hairless mice, keratinocytes, fibroblasts	Prevents photoisomerisation of *trans*-UCA (counteracting immunosuppression) and increases antioxidant activity. Reduces DNA lesions, proliferation and the inflammatory response.	Mice: Reduces AK occurrence, delays tumour onset and reduces tumour incidence.	[124,125,126,127,128,129,130,131,132,133,134,135,136]
Pomegranate	Healthy volunteers, hairless mice	Reduces DNA lesions, oxidative damage, the inflammatory response and signal transduction.	Mice: Reduces SCC occurrence.	[139,140,141,142]
Pterostilbene	Hairless mice, keratinocytes	Reduces oxidative stress via increased antioxidant capacity.	Mice: Reduces tumour incidence and multiplicity.	[122]
Raspberries	Hairless mice, fibroblasts	Reduces oxidative DNA damage and stress via increased antioxidant activity. Reduces the inflammatory response and AP-1 and NF-κB activity.	Mice: Reduces tumour size and multiplicity.	[143,144,145]

Abbreviations: AK: actinic keratosis, AP-1: activator protein 1, IL: interleukin, SCC: squamous cell carcinoma, UCA: urocanic acid.

## 4. Vitamins and Derived Compounds 

### 4.1. Vitamin A: The Retinoids 

Vitamin A represents a class of naturally occurring or synthetically produced compounds that share structural or functional properties and are commonly referred to as retinoids. Dietary sources of vitamin A include plant products containing provitamin A carotenoids [156] or animal products such as dairy products, eggs and liver in which vitamin A is present as retinol, retinal, retinyl esters and retinoic acid (RA) [157,158]. Because the photoprotective potential of carotenoids has been explored in other reviews [159], the following description will focus on the latter group.

In vitro studies have shown that retinoids have antioxidant activity that prevents ROS formation, in part due to restoration of Nrf2 [160]. Furthermore, retinoids exhibit anti-inflammatory properties by reducing AP-1 activity [161,162] and TNF-α expression [163,164]. However, because topical retinoid treatment can cause skin irritation [165,166], the elicited inflammatory responses must be considered in their specific contexts.

Whereas topical application of retinoids protects against DNA damage and apoptosis in hairless mice [167], the effects on photocarcinogenesis reportedly vary and remain controversial. Two separate experiments from the same group led by Epstein et al. showcased these opposing effects of RA, reporting stimulation of photocarcinogenesis in one study [168] but inhibition in another [169]. A similar paradox has been reported by other groups, with stimulatory [166,170,171], inhibitory [172], and no effect on photocarcinogenesis in hairless mice treated with RA all being described [173]. No mechanism has been suggested that can resolve these conflicting results. 

Consequently, few human studies have been performed. One study reported that the application of a 2% retinyl ester cream protected against thymine dimers and erythema in healthy volunteers 24 hours after UVR treatment [174]. However, topical application of a 0.05% all-trans RA cream for eight days had no effect on MED [175]. Although this discrepancy may be caused by differences in the retinoic compounds or their concentrations, it may also reflect the pattern observed in the mouse studies. 

Despite this, a recent cohort study reported that increased dietary intake of retinol led to a decreased risk of SCC [176]. Similarly, in psoriasis patients exposed to psoralen-UVA, systemic retinoid use was associated with a significant reduction in SCC risk [177]. In a trial focusing on subjects with a history of AKs, a dietary supplement of 25,000 IU retinol over five years protected against SCC but not BCC incidence (Table 4) [178]. However, the same group reported a lack of effect in high-risk subjects with a history of KCs [179]. Taken together, these results indicate that we still do not know enough about how retinoids function and whether they can consistently provide photoprotection. 

### 4.2. Vitamin B3: Nicotinamide

Vitamin B_3_ represents a family of water-soluble compounds with similar structures that include nicotinamide, nicotinic acid and nicotinamide riboside. These are found in fish, meat and wheat, with smaller quantities present in vegetables. Vitamin B_3_ compounds act as precursors for the cofactor nicotinamide adenine dinucleotide (NAD^+^) which is involved in ATP metabolism [180,181,182]. NAD^+^ deficiency increases the skin’s sensitivity to UVR exposure by reducing genomic stability and preventing DNA repair [183]. Therefore, replenishing NAD^+^ precursors is a potential strategy in cancer prevention. 

NAD^+^ acts as a substrate for poly ADP-ribose polymerase-1 (PARP-1) and the sirtuin proteins that regulate DNA repair and genomic stability [184,185]. Incubation of irradiated keratinocytes with nicotinamide enhanced DNA repair and reduced photolesions [186]. Furthermore, pre-treatment also prevented UV-induced inflammation in keratinocytes by reducing the expression of IL-6, IL-10, TNF-α and COX-2 [187,188]. 

Gensler et al. have described the effect of nicotinamide in mouse models and found that topical and systemic administration resulted in decreased tumour incidence in irradiated BALB/c mice [189,190]. Moreover, these mice exhibited reductions in tumour development, tumour multiplicity and UV-induced immunosuppression.

Topical application of a 5% nicotinamide cream also reduced immunosuppression in healthy human volunteers [191,192]. As an oral delivery study reported similar protective results [193], with no effect on MED following topical application [191], the effects of nicotinamide are likely caused by protection against UV-induced events rather than sun-screening (Table 4). 

The effects of treating sun-damaged individuals with nicotinamide were investigated in a phase II double-blinded randomised controlled trial. In volunteers with ≥4 palpable AKs, 500 mg dietary supplement reduced the mean AK count over a period of four months (21.6 vs. 34.8 in the control group) [194]. Similar effects were reported over one year in a phase III trial involving high-risk subjects, with dietary supplements reducing the rate of new KCs (1.8 per person in nicotinamide-treated subjects vs. 2.4 in control subjects) and SCCs (0.5 vs. 0.7 per person, respectively) [195]. This trial reported few adverse effects and no additional effect at higher doses, suggesting that 500 mg is a safe and effective dose. However, no beneficial effects were observed on recurrent carcinomas or following treatment discontinuation [195]. 

The phase III trial was notable for an increase in infections among the treated group and significant differences in the numbers of skin and mucocutaneous infections [196]. Although the authors accepted that all adverse effects should be considered, they also noted that nicotinamide reportedly increases the clearance of skin infections [197,198]. Nevertheless, Yélamos et al. noted that while an overall reduction in KC rates was observed following nicotinamide intake, the more aggressive types of SCCs and BCCs apparently increased [199]. 

Therefore, although nicotinamide has protective effects in human studies, additional trials with a focus on patient follow-up and the differences among and subtypes will be necessary to address these concerns. 

### 4.3. Vitamin C

Vitamin C is a key antioxidant found in the skin where it plays a role in keratinocyte viability and maintenance of the epidermal barrier [200]. Vitamin C can be synthesised from D-glucose in plants and almost all non-primates [201], whereas humans can no longer produce the active enzyme required for this process. Therefore, humans obtain vitamin C from their diet. 

The antioxidant properties of vitamin C are well established. In keratinocytes, treatment with vitamin C reduced ROS formation [202], oxidative DNA damage [203] and LPO, simultaneously preventing glutathione depletion [204]. Treatment with vitamin C also affects inflammatory responses, leading to decreased expression and release of IL-1α, IL-6, IL-8 and TNF-α [202,204,205], while also preventing apoptosis and MAPK activation (Table 4) [202,206]. 

Despite these effects and its role as an antioxidant, the photoprotective potential of vitamin C is less clear in in vivo studies. In a study performed in 1982, dietary supplementation of vitamin C given to hairless mice led to a delay in UV-induced tumour onset and decreased tumour incidence [207]. A second study, performed nine years later, confirmed these observations [208]. However, in 2005, a study involving a similar set-up observed an increase in UV-induced tumour multiplicity [93], indicating some uncertainty regarding the effect of vitamin C when more complex models are used. 

Furthermore, few clinical trials have investigated the photoprotective effects of vitamin C. In a study performed in 2002, 500 mg oral supplement of vitamin C over an eight-week period had no effect on the UV-induced erythemal response measured [209]. Studies exploring higher doses of 2 g and 3 g—albeit over shorter periods (7 and 50 days, respectively)—also reported no significant effects [210,211]. Topical application of a 5% cream also offered no protection in healthy volunteers [212,213], and cohort studies lasting more than ten years have found no evidence that vitamin C intake decreases the incidence of BCCs and SCCs [214,215]. 

Because vitamin C alone does not appear to provide photoprotection in vivo, the focus of research has shifted to investigating vitamin C combined with other compounds. Combining vitamins C and E has produced promising clinical results and is further described in Section 4.5 [211,216,217]. Similarly, a topical formulation of vitamin C, ferulic acid and the phenolic compound phloretin conferred protection against UV-induced erythema, apoptotic sunburn-cell production and thymine dimer formation in healthy volunteers [218], indicating that vitamin C in synergy with other compounds may provide sufficient protection. 

### 4.4. Vitamin D_3_

The epidermis is the primary source of vitamin D_3_ (cholecalciferol). Vitamin D_3_ is a fat-soluble steroid hormone primarily obtained by de novo synthesis in the skin with only a fraction obtained through the diet. In the skin, a cholesterol precursor is converted to pre-vitamin D_3_ and then to active vitamin D_3_ (1,25(OH)_2_D) in a reaction catalysed by sunlight [219]. Despite this reliance on a major source of carcinogenesis, increasing evidence suggests that vitamin D_3_ may act as a tumour suppressor via crosstalk with p53 [220]. 

Expression of p53 is increased in irradiated keratinocytes following 1,25(OH)_2_D treatment [221,222]. Incubation with 1,25(OH)_2_D or analogues with low calcaemic activity also protected against cell death and CPD formation [223,224], while 1,25(OH)_2_D and a non-genomic analogue provided protection against CPDs and 8-oxo-Gua lesions in ex vivo studies [225].

In in vivo studies, topical administration of 1,25(OH)_2_D, as well as low calcaemic and non-genomic analogues, protected hairless mice against immunosuppression and DNA damage, inducing p53 expression to promote cell cycle arrest [222,226]. In addition, 1,25(OH)_2_D and the non-genomic analogue reduced photocarcinogenesis, decreasing tumour incidence and multiplicity as well as reducing the number of tumours that progressed to SCCs [222]. Another study demonstrated similar protective effects but by week 26, the 1,25(OH)_2_D-treated mice exhibited more than 20% weight loss, reportedly due to chronic hypercalcaemia [227]. This side effect was also noted in the aforementioned study and was addressed by decreasing the concentration of 1,25(OH)_2_D administered [226]. 

A recent clinical trial investigated the effect of vitamin D_3_ as a photoprotectant in human studies. This study included doses such as 50,000, 100,000 and 200,000 IU, with only the latter reportedly capable of protecting against UVR-induced oedema and decreasing TNF-α expression. A clustering analysis identified one distinct cluster primarily containing participants who received the highest dose. The participants in this cluster had increased serum vitamin D_3_, reduced erythema and a distinct gene profile when compared to another group of participants, many of whom received placebo [228]. Vitamin D_3_ protected against upregulation of pro-inflammatory mediators and induced genes involved in skin barrier repair, enhancing photoprotection (Table 4). Furthermore, this study reported no adverse effects or increases in serum calcium, suggesting that the vitamin D_3_ dose used was safe for testing in future studies. 

The mechanism underlying vitamin D_3_ photoprotection is not entirely clear. Mice, where the vitamin D receptor (VDR) has been knocked out, are more prone to photocarcinogenesis [229]. Because VDRs with mutations in the DNA binding domain are still capable of stimulating photoprotection [230], this may be mediated through a non-genomic pathway. Consequently, although vitamin D_3_ has protective effects, further studies are needed to determine its mechanism of action and effect on human carcinogenesis. 

### 4.5. Vitamin E: α-Tocopherol

Vitamin E covers a class of fat-soluble compounds, the tocopherols and tocotrienols. α-tocopherol has the greatest biological activity and is the most abundant form of vitamin E in the skin. Plants can produce vitamin E to protect against sunlight, whereas humans cannot and therefore rely on leafy greens, vegetables and nuts to provide vitamin E through the diet [231]. Similar to vitamin C, vitamin E intake had no protective effect on KC incidence [214,215]. Despite this, vitamin E did exhibit several noteworthy photoprotective properties.

Vitamin E functions as an antioxidant by scavenging radicals to reduce the damaging effects of oxidative stress [232,233,234]. Incubation with vitamin E protected keratinocytes against UV-induced cytotoxicity, apoptosis and NF-κB activation [235,236] Moreover, incubation with vitamin E before or after UVR exposure reduced the appearance of CPDs and oxidative DNA lesions [233], indicating that these observations were not solely attributed to a sun-screening effect (Table 4). 

In hairless mice, topical application of vitamin E reduced UV-induced erythema and oedema [237,238], as well as immunosuppression and tumour incidence [239]. Similarly, dietary vitamin E supplementation resulted in delayed tumour onset and reductions in tumour multiplicity and size. These mice also exhibited decreased proliferation and oxidative stress markers such as 8-oxo-Gua lesions [240]. However, a more recent study performed in 2013, reported contrasting effects with a topical vitamin E cream increasing tumour formation and proliferation, DNA damage and angiogenesis [241]. 

One clinical trial reported that 400-IU vitamin E dietary supplementation over a six-month period did not significantly change MED or sunburn-cell formation [242]. A shorter trial with the same dose over an eight-week period did not report any protective effects for vitamin E either [243]. 

Although neither vitamin C nor E showed significant photoprotection in human studies, combining the two vitamins has promising effects [244]. Adding ferulic acid to a combined preparation of topically applied vitamins C and E improves the stability and doubles the photoprotective capacity of the formulation when applied to pigskin, as measured by thymine dimer formation, erythema and apoptosis [245]. In a clinical trial spanning eight days, dietary supplementation of vitamins C and E increased MED (median of 80 mJ/cm^2^ before supplementation to 96.5 mJ/cm^2^ after eight days) [217]. Similar increases in MED, as well as reduced thymine dimer formation, were observed in longer trials with no effect for the vitamins separately [211,216]. Overall, these results indicate that vitamins C and E can act synergistically to protect against UVR exposure.

**Table 4:** Selected vitamins and derivates that have been tested for photoprotective effects, their proposed mechanisms and, if applicable, (pre-)clinical results.

Compound	Model	Mechanism of Action	(Pre-)Clinical Results	Ref
Vitamin A: Retinoids	AK- and KC-affected patients, healthy volunteers, hairless mice, fibroblasts	Reduces DNA damage and the inflammatory response via AP-1 and NF-κB inhibition. Reduces oxidative stress by inducing Nrf2.	Human: Reduces SCC risk *. Mice: Delays tumour onset and reduces tumour incidence **.	[160,161,162,163,164,167,169,172,174,177,178]
Vitamin B_3_: Nicotinamide	AK-affected and high-risk patients, hairless mice, keratinocytes	Induces DNA repair by acting as an NAD^+^ precursor. Reduces immunosuppression and the inflammatory response.	Human: Reduces AK occurrence and rate of new KCs and SCCs Mice: Reduces tumour incidence and multiplicity.	[186,187,188,189,190,191,192,193,194,195]
Vitamin C	Healthy volunteers, hairless mice, keratinocytes	Increases antioxidant capacity and reduces ROS formation, DNA lesions and LPO. Reduces the inflammatory response.	Mice: Delays tumour onset and reduces tumour incidence **.	[202,203,204,205,206,207,208]
Vitamin D_3_	Healthy volunteers, hairless mice, keratinocytes, ex vivo skin	Non-genomic signalling via the VDR facilitates protection against DNA lesions and cell cycle arrest while reducing immunosuppression.	Mice: Reduces tumour incidence, multiplicity and progression.	[221,222,223,224,225,226,227]
Vitamin E: α-tocopherol	Healthy volunteers, hairless mice, keratinocytes	Prevents oxidative damage by increasing antioxidant activity. Reduces DNA damage, immunosuppression, proliferation, apoptosis and the inflammatory response via AP-1 and NF-κB inhibition.	Mice: Delays tumour onset and reduces tumour incidence, multiplicity and size **.	[233,234,235,236,237,238,239,240]

*: inconsistent results, **: contrasting results have been reported (retinoids [166,170,171]; vitamin C [93]; α-tocopherol [241]). Abbreviations: AK: actinic keratosis, AP-1: activator protein 1, KC: keratinocyte carcinoma, LPO: lipid peroxidation, NRF2: nuclear factor erythroid 2-related factor 2, ROS: reactive oxygen species, SCC: squamous cell carcinoma, VDR: vitamin D receptor.

## 5. Perspectives and Concluding Remarks 

When sun-avoidant behaviour, protective clothing and sunscreens are used insufficiently to prevent KCs, oral photoprotection presents a promising alternative or supplement. Instead of blocking the absorption of UV-rays, dietary intake of protective compounds focuses on preventing and countering UV-induced events that stimulate photocarcinogenesis (Figure 1 and Figure 2). Using oral photoprotectants in addition to sunscreen will increase the protection against UVR-induced effects. 

Drug repurposing provides a promising avenue for systemic photoprotection, with varying degree of success in pre-clinical trials (Table 2). However, applying these findings to clinical trials may be difficult as data obtained from studies in unrelated conditions may not be relevant for evaluating the efficacy and safety of treatments to prevent photocarcinogenesis in humans. Therefore, new investigations are needed to identify therapeutic windows in which photoprotection is effective. 

Phytochemicals are promising photoprotectants. Their protective effects (Table 4), low toxicity and widespread availability make them ideal candidates for systemic photoprotection. Nevertheless, not all compounds are effective via ingestion, and phytochemicals may require extraction, purification and concentration to generate effective photoprotective products. Furthermore, some phytochemicals may only be effective via topical administration if they exert their effects by absorbing UVR, making them unsuitable for systemic photoprotection.

Because alternatives to conventional treatments are being considered, it is important to remember that photoprotection requires a degree of individualisation: side effects that are unacceptable to some individuals may be tolerable to others, if the treatment decreases the risk of carcinogenesis. Thus, phytochemicals with photoprotective properties could be administered in safe and effective doses to less affected individuals where milder strategies may still provide an effect. Whereas patients receiving immunosuppressive treatments who have a higher risk of developing KCs should be treated with more aggressive pharmaceutical therapies to ensure efficient treatment of the carcinomas. 

Photoprevention in its current stage is focused on preventing the initial steps of KC development by counteracting the adverse effects induced by UVR exposure. However, increasing evidence has demonstrated that phytochemical supplementation to cancer treatments such as chemotherapy may improve treatment outcome. In chemotherapy, natural or synthetic compounds such as bleomycin, cisplatin and taxol induce cytotoxicity in tumour cells by interfering with DNA replication and mitosis to prevent tumour proliferation. The addition of certain phytochemicals to these treatments have demonstrated better outcomes, either by improving treatment efficacy, drug delivery and accumulation [246] or improving management of side effects in in vitro and pre-clinical models [247], which is further reviewed here [248]. 

In general, more studies are needed to clarify whether photoprotectants on their own can be used as anticarcinogenic therapies, as well as to identify the most promising targets for photoprotection. Because these compounds may display weak pharmacological potencies, efficient use may require modification either through synthetically edited structures or the use of adjuvants to improve results. As the compounds presented in this review affect several different targets, optimisation of their cellular effects must also be considered. Signal transduction is a delicate process evolved to respond to different stimuli resulting in unexpected results that can both promote and prevent cellular growth. As such, treatment with these compounds must be optimised through comprehensive studies to ensure that the induced outcome is predominantly anticarcinogenic.

**Figure 3:**
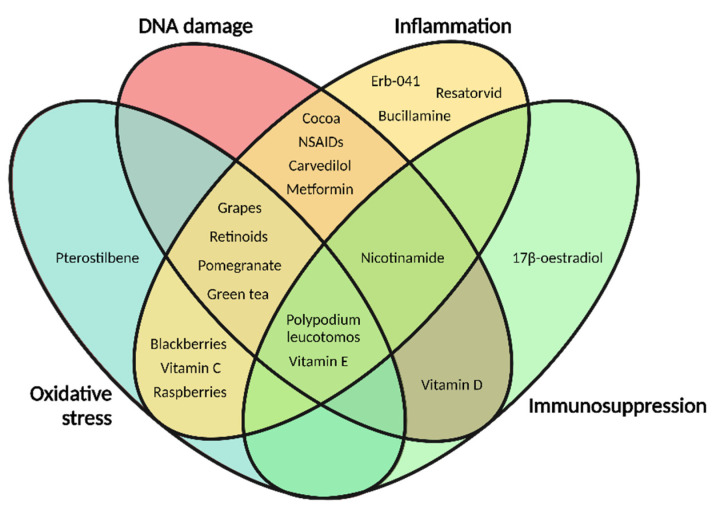
Overview of the reviewed compounds and their mechanisms. The Venn diagram represents four of the five UV-induced events (DNA damage, oxidative stress, inflammation and immunosuppression). UV-induced events targeted by each compound are shown. Dysregulated signal transduction is not included in the figure as it can be reflected as induction and stimulation of the other four UV-induced events. Abbreviations: NSAIDs, non-steroidal anti-inflammatory drugs.

In this review, we have discussed the photoprotective potential of 15 different categories across pharmaceuticals, phytochemicals and vitamins (Table 2, Table 3 and Table 4). We have also provided an overview of the current understanding of the UV-induced events they target under the headings of DNA damage, oxidative stress, inflammation, immunosuppression and dysregulated signal transduction, as summarised in Figure 3. 

Numerous of studies have explored the extensive possibilities of photoprotectants, and through thorough investigations focusing on the various mechanisms of action following oral delivery, the challenge will now be to identify the most promising candidates to provide the best possible photoprotection to prevent skin cancer.
